# Microbiological Analysis of Surfaces of Leonardo Da Vinci's *Atlantic Codex*: Biodeterioration Risk

**DOI:** 10.1155/2014/214364

**Published:** 2014-12-10

**Authors:** Gianfranco Tarsitani, Catia Moroni, Francesca Cappitelli, Giovanna Pasquariello, Oriana Maggi

**Affiliations:** ^1^Dipartimento di Scienze Medico-chirurgiche e Medicina Traslazionale, Sapienza Università di Roma, Piazzale Aldo Moro 5, 00185 Rome, Italy; ^2^Dipartimento di Sanità Pubblica e Malattie Infettive, Sapienza Università di Roma, Piazzale Aldo Moro 5, 00185 Rome, Italy; ^3^Dipartimento di Scienze per gli Alimenti, La Nutrizione e l'Ambiente, Università degli Studi di Milano, Via Celoria 2, 20133 Milan, Italy; ^4^National Institute of Graphic Arts, MIBACT, Via della Stamperia 6, 00187 Rome, Italy; ^5^Dipartimento di Biologia Ambientale, Sapienza Università di Roma, Piazzale Aldo Moro 5, 00185 Rome, Italy

## Abstract

Following the discovery of discoloration on some pages of the *Atlantic Codex (AC)* of Leonardo da Vinci kept in the Biblioteca Ambrosiana in Milan, some investigations have been carried out to verify the presence of microorganisms, such as bacteria and fungi. To verify the presence of microorganisms a noninvasive method of sampling has been used that was efficient and allowed us to highlight the microbial facies of the material that was examined using conventional microbiological techniques. The microclimatic conditions in the storage room as well as the water content of the volume were also assessed. The combined observations allowed the conclusion that the discoloration of suspected biological origin on some pages of *AC* is not related to the presence or current attack of microbial agents.

## 1. Introduction

The* Atlantic Codex* (*AC*) is the largest collection of drawings and writings by Leonardo da Vinci, including 1,119 pages collected in 12 volumes, and it is currently preserved at the Biblioteca Ambrosiana in Milan. The sheets (64.5 × 43.5 cm) were assembled in no particular order and cover a long period of Leonardo studies, from 1478 to 1519. The drawings and writings focus on different topics: anatomy, astronomy, botany, chemistry, geography, mathematics, mechanics, machinery drawings, studies on the flight of birds, and architectural projects. The* AC* has undergone several restorations and the new binding in 12 volumes occurred in the period 1970–73. In relation to the present assessments, that restoration period is probably the critical starting time of the discoloration when one considers that Leonardo da Vinci's antique pages were pasted on modern paper at that time. Following the discovery of some discoloration on the pages of the* AC*, some investigations have been carried out. In October-November 2007, the* AC* was fully digitized and since 2008 several analyses and assessments have followed, including the present study, to estimate the presence of bacteria or microfungi possibly contributing to ongoing damage.

In the old maps drawn by Leonardo the raw materials were mainly composed of selected cotton cloth containing almost pure cellulose [1]. The biological attack of paper materials is therefore mainly due to cellulolytic organisms such as bacteria and fungi [2–6]. The presence in these organisms of the cellulase enzyme complex can catalyze specific actions to break the polymer. The damage varies from erosion to formation of age spots more or less pronounced [7, 8].

The biological attack is a function of environmental relative humidity and correlated levels of water absorption [9]. Relative humidity is a function of both the absolute amount of water present in the air and ambient temperatures. When the materials reach water content greater than 10% (usually because the air relative humidity is above 60%), some species can germinate and grow [10]. The water demand varies significantly more or less depending on the organisms, which can be defined as* hydrophilous* or* xerophilous* [11–13].

The research was carried out on some antique* AC* pages to determine the nature of the discoloration, a suspected biotic origin, and the possible presence of hazardous conditions for the precious manuscript, using both culture and culture-independent microbiological techniques [14–16].

## 2. Materials and Methods

### 2.1. Sampling

**Figure 1:**
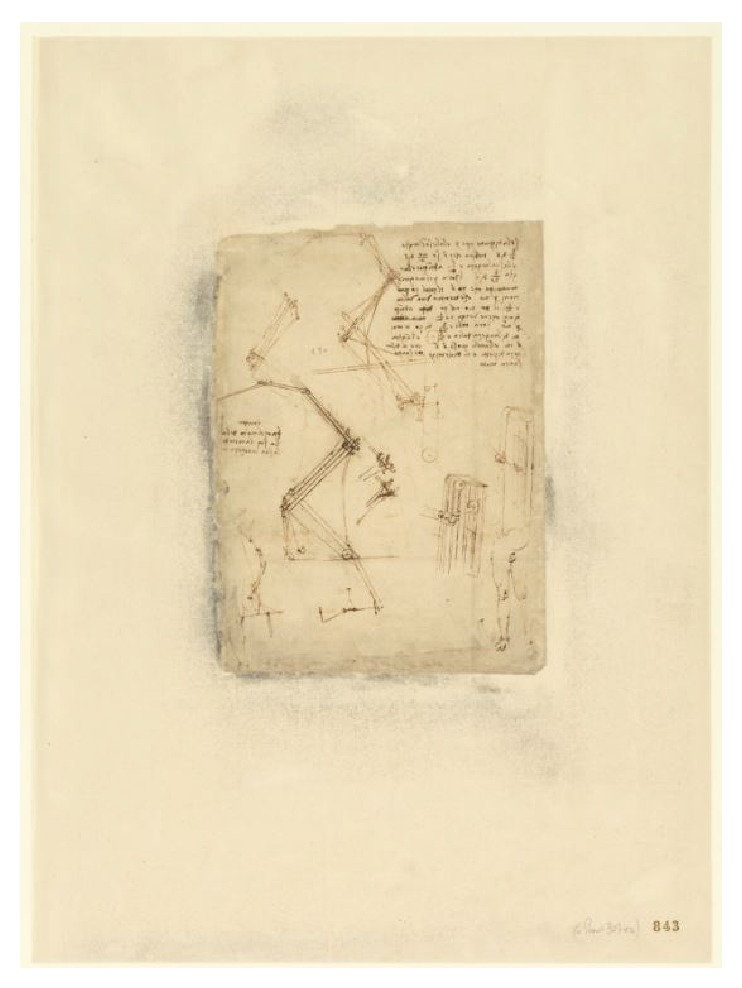


The sampling campaigns were carried out in two different days spaced about one month in June and in July. The first intervention included visual and instrumental observation (handle magnifying lighted glass—10x magnification) of the antique sheets as they were affixed onto new pages and the taking of samples, using a noninvasive method. In particular, the original (antique paper—on* AC*) and new support sheets (modern paper—outer* AC*) pages 673, 776, and 843 (Figure 1) of the* AC* were tested, both in areas stained and not stained (on and outer* AC*), for microbiological culture and molecular analysis. Some control samples were done on one facsimile of the* AC* (commercial scanned copy) kept in an environment next to the* AC* repository.

During the second intervention, the percentage of water in the pages of the* AC* was evaluated; the spots were observed with the help of an optical microscope (60x–100x lighted mini-microscope) and microbiological samples of pages 843 and 895 (verso and recto) were taken.

### 2.2. Microclimatic Measurements

The microclimatic parameters of temperature and humidity were recorded with punctual measurements during the two days of sampling with the use of thermohygrometer Salmoiraghi. Water content (%) of the pages of the* AC* was measured by the contact hygrometer Aqua Boy with a fork-shaped probe.

### 2.3. Cultivation Assays

To verify the presence of microorganisms on the pages of the volume a noninvasive sampling technique was used [17]. The aseptic sampling was performed by a single operator using nitrocellulose membranes (Sartorius AG, Göttingen, Germany, 47 mm in diameter, corresponding to an area of 17.34 cm^2^), handled with sterile forceps on the surface of the manuscript, gently pressed with a sterile swab for 30 s. Then the surfaces of membranes were rubbed repeatedly with a sterile cotton swab, electrostatically charged to improve the adhesion of particles present. Thereafter, the membranes were laid out on RODAC contact plates of 55 mm in diameter, containing the following media: Tryptone Soy Agar (TSA) for the mesophilic bacteria; Sabouraud Agar + chloramphenicol (SAB) for the microfungi; Mannitol Salt Agar (MSA) for staphylococci. Petri dishes were incubated at 37°C for 48 hours to assess the microbial loads in TSA and MSA. SAB dishes were placed at 28°C for 7 days, and data were reported as Colony Forming Units (CFU)/m^2^.

The fungal strains were isolated in pure culture using media suitable for each taxonomical group [18] and placed at 25°C (CYA: Czapek Yeast Agar, MEA: Malt Extract Agar, PDA: Potato Dextrose Agar, and MEA: Malt Extract Agar).

### 2.4. Molecular Identification of the Bacterial Isolates

Bacterial isolates were identified by 16S rDNA sequencing using the primers pair 27F and 1495R [19]. Total genomic DNA was extracted as reported by Polo et al. [20]. Amplification of the nucleic acid was conducted in a 50 *μ*L reaction volume consisting of 1X PCR polymerase chain reaction (PCR) Rxn buffer, 1.5 mM MgCl_2_, 200 nM of dNTP mix, 250 nM each of the forward and reverse primers, 2.5 U of Taq DNA polymerase (Invitrogen), and 1 *μ*L DNA template. The thermal cycling programme included an initial denaturation at 95°C for 2 min followed by 5 cycles consisting of denaturation at 95°C for 30 s, annealing at 60°C for 30 s, and extension at 72°C for 4 min, 5 cycles consisting of denaturation at 95°C for 30 s, annealing at 55°C for 30 s, and extension at 72°C for 4 min, 20 cycles consisting of denaturation at 94°C for 30 s, annealing at 50°C for 30 s, and extension at 72°C for 4 min, and a final extension step at 72°C for 10 min. Aliquots of amplicons were loaded in 1.2% agarose gel in 0.5X TBE buffer (45 mM Tris-borate, 1 mM EDTA) to verify specificity. Amplicons were sequenced at an external service (Primm, Milan, Italy). The sequences were analysed using the BLASTN software (http://www.ncbi.nlm.nih.gov/BLAST, Lane [19]; Polo et al. [20]). 

The fungal identification was made by means of morphological analysis, using the above mentioned culture media.

## 3. Results

From the microscopic observations of pages 673, 776, 843, and 895 it was possible to detect certain characteristics of these coloured spots. They consist of a set of small dark green-gray jagged spots. Their aspect did not appear to be due to presence of typical microbial indicators. The paper of the pages examined (modern paper), in particular at locations where they are glued to Leonardo's antique pages (especially in areas to the interior of the volume), shows blisters (convex) and craters (concave), features that do not seem to be present on the antique sheets of paper. Furthermore, in recent months, the observation of page 843 with a magnifying glass and comparison with the scanned image enlarged in 2007 has revealed that the discoloration in question has not enlarged.

In the first inspection the air temperature and humidity detected in the conservation environment were 19°C and 54%; in the second survey 20°C and 47%, these values fall within the range chosen to calibrate the air-conditioning (19.5 to 20.5°C and 40–55% RH).

The water content (%) of the* AC* pages was found to be 8% in samples taken on the first sheet, close to the binding, and 10% in the inner pages of the manuscript, then close to the lower limit of the values that may pose a risk of microbial growth. The microbiological results carried out on pages 673, 776, 843, and 895 showed the presence of modest and unremarkable average contamination <3 CFU/m^2^ for bacteria and <2 CFU/m^2^ for microfungi both on the surfaces that have stains and those with no stains. As for microfungi, the number of CFUs isolated from the* AC* (Table 1) was 36, with 5 genera (*Alternaria*,* Aspergillus*,* Eurotium*,* Paecilomyces*, and* Penicillium*) represented by 12 species and a superior taxon (*Saccharomycetaceae*).

**Figure 2:**
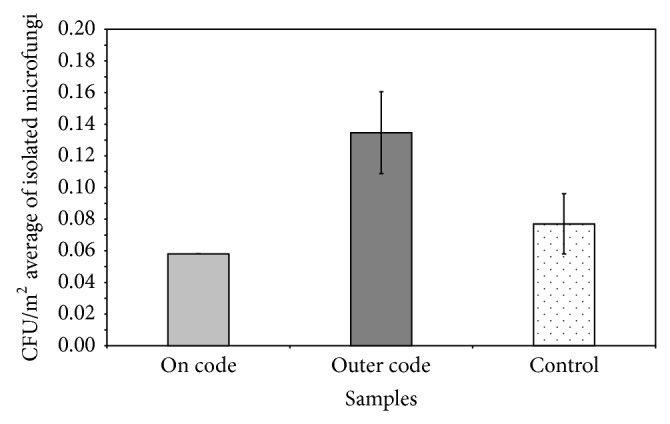
CFU/m^2^ average of microfungi isolated from the two sampling campaigns, on Code, outer Code, and control.

Figure 2 shows the average CFU/m^2^ of microfungi isolated during the two sampling campaigns on the* AC*, on the outer* AC* and in the control, ranging from 0.06 to 0.14, with higher values for the samples taken outside of the* AC*. Overall, these recorded values are still very low.

The analysis of the sequences obtained from isolated bacteria (Table 2) has revealed the presence of the following taxa:* Staphylococcus hominis*,* S. epidermidis* and* Staphylococcus* spp.,* Massilia timonae*,* Brevundimonas vesicularis*,* Bacillus muralis* and other* Bacillus* spp.,* Micrococcaceae*, and* Spiroplasmataceae*. Our data reveal that the microorganisms identified by sequencing of isolated bacteria from sheets of ancient and modern paper are similar. These sequences are very different from the facsimile of the* AC* used for the control.

## 4. Discussion

The studies undertaken of the stains seen on some pages of the* AC* reveal that they do not seem to be caused by microbial growth. From these observations it could be assumed that the discoloration is attributable to a degradation of the adhesive used in the last restoration (1970–1973). Over time, this degradation may have been induced by the interaction of a number of exogenous factors such as variations in temperature and humidity [14, 16, 21–23], the presence of chemical and biological pollutants, dust, and endogenous factors (chemical and physical characteristics of the media-sheets of paper and originals). The careful observation of the stained pages compared with the magnified scanned image can affirm that, in this last year, the chromatic alteration in question has not progressed.

The results of microbiological research revealed a widespread presence of vital microorganisms of environmental and human origin. Different species have been identified as potential biodeteriogen microfungi for paper materials [6], including some which can cause allergies or be pathogenic to humans [24, 25], but the microbial loads are not significant for the possibility of a biodeteriogenic attack. These data are accompanied by the detection of microclimatic data compatible with a good preservation of manuscripts, while the humidity of the paper in the two sampling campaigns was once normal and the other at the lower limit for risk of microbial growth.

Among isolated bacteria, opportunistic pathogens of* Staphylococcus* were identified, as well as* Massilia timonae* [26] and* Brevundimonas vesicularis* [27]. Other bacteria found included* Bacillus muralis*, a novel species isolated for the first time from mural paintings located in Spain (necropolis of Carmona) and Germany (church of Greene-Kreiensen) [28];* Bacillus* spp., spore-forming bacteria found in a wide range of habitats; and* Spiroplasma*, shown to be associated with arthropods [29].

The genus* Aspergillus* is the taxon with the highest number of CFU in the three samples. From Table 2 it is also clear that the genus* Aspergillus* is represented by the greatest number of species (6) in the three samples, and among these species* A. flavus*,* A. fumigatus*,* A. niger*,* A. terreus*, and* A. versicolor* are those which are found more often in books and documents, representing a potential risk of degradation of the support materials if stored in inappropriate conditions [30–33]. It is also known that the longevity of their spores can range from 2 to 20 years [8, 34]. These species together with* Eurotium repens* and* E. amstelodami* can also be harmful to humans, for their ability to cause pulmonary invasive aspergillosis, cutaneous infections, keratitis, and allergic reactions [24, 35, 36].* Paecilomyces variotii* is a fungal species commonly found in storage areas of libraries and archives [37].* Penicillium chrysogenum* and* Alternaria alternata* are known as paper biodeteriogens [23] and* Penicillium expansum* was found capable of degrading straw-cellulose [38].

Significant differences in the development of microorganisms were not observed from samples taken on the specified pages with and without discoloration on “modern” support paper and antique sheet of paper and on stained and unstained areas.

Analogous to what is found with conventional microbiological techniques, molecular analysis conducted on the whole bacterial and fungal communities by Principi et al. [39] confirmed the indifference of bacterial and fungal species distribution with respect to the observed spots.

The critical issues that are revealed by microbiological studies are twofold: the presence of staphylococci in different samples was correlated with human skin contamination and may be the stimulus to improve handling procedures for consultation of the manuscripts; the presence of several microfungi, potential biodeteriogens for paper material and pathogens for humans, indicates the need to keep the microclimate under control. Situations of oscillation parameters may induce the development of potential biodeteriogens of paper material that are present in the air and/or dust. In this regard it is worth mentioning that the inside pages of the* AC* had a percentage of water at the limit of acceptability (10%). This value can be said to represent the “threshold of biological risk” that must not be exceeded. The biodeterioration phenomenon is an integrated system of factors, the result of the interaction between the presence and activity of biodeteriogen microorganisms in the environment, microclimatic parameters, and the chemical and physical properties of various materials in the manuscript. Biodeterioration then occurs under certain environmental conditions: relative humidity greater than 60%, temperatures above 20°C, and percentage content of water in excess of 8–10%.

The vault in which the* AC* is stored will keep it secure and is designed to ensure proper storage temperature and relative humidity. The critical environmental conservation can be derived possibly from the promiscuity of the things stored in the large space dedicated to not only the preservation of* AC,* but also other materials.

## 5. Conclusion

Despite the fact that the* Atlantic Codex* presented some damage, the set of observations indicate that the discoloration of suspected biological origin on some of Leonardo da Vinci's antique pages is not directly related to the presence of microorganisms and that, currently, elements indicative of biodeterioration risk of microbiological origin are very limited. Other observations include the following: the structure of the discoloration does not seem typical of microbial growth; based on accurate monitoring of stained pages over time, it can be said that the chromatic alteration has not progressed in the past year; the proper storage temperature and relative humidity are present in the vault where the* AC* is preserved; and there is an absence of significant differences in the development of microorganisms in samples taken from traditional microbiological cultures on pages with and without discoloration.

This first important conclusion that derives from the confluence of several observations, however, should not obscure the risks to which the precious ancient manuscript is exposed. Problems were detected at different levels, namely: the mixing of the materials kept in the storage room that do not offer the absolute environmental hygiene; the presence of staphylococci in various microbiological samples indicates skin contamination through handling of the manuscript; the presence of different microfungi, potential biodeteriogens for paper material and pathogens for humans. Finally, it is worth mentioning that the internal pages have presented the percentage of water to the limit of acceptability (10%). The results of the study are reassuring about the state of conservation of the manuscript in that there are no vital signs of attack by cellulolytic biodeteriogen microorganisms, but the results also indicate the need to monitor continuously and with great accuracy both the microclimate of the storage room to keep the conditions sterile and the mode of consultation of the* AC*. Regarding the genesis of the stains that were examined, one cannot reject the hypothesis that their origin may have been biological, perhaps borne of the glue used in the restoration. Finally it can be briefly said that the studies show that it is not currently in place a biological attack on the precious AC.

These results highlight two general observations. The first is that the restoration techniques need to be continually revisited and revised in the light of scientific evidence. The second, closely related to the first, is that we must strengthen the observations and research in the sphere of conservation and restoration. We also believe that the use of molecular techniques in combination with conventional methodologies should be adopted in the field of cultural heritage diagnostics.

## Figures and Tables

**Table 1:** Species and CFU of isolated microfungi from sampling campaigns (1st and 2nd) on Code, outer Code, and control.

	On Code	Outer Code		Control		Total
	1st	1st	2nd	1st	2nd	
*Alternaria alternata *		1				1
*Aspergillus flavo *				1		1
*Aspergillus fumigatus *	1					1
*Aspergillus nidulans *				1		1
*Aspergillus niger *	1		1			2
*Aspergillus terreus *		8	7		1	16
*Aspergillus versicolor *		4			1	5
*Eurotium amstelodami *	1					1
*Eurotium repens *			1			1
*Paecilomyces variotii *			1			1
*Penicillium chrysogenum *			2			2
*Penicillium expansum *		2				2
*Saccharomycetaceae *		1				1
*Mycelia sterilia hyaline *	1					1

Total CFU	4	16	12	3	1	36

**Table 2:** 16S rDNA-based identification of bacterial isolates. M: modern paper, A: antique paper.

Page	Type of paper	Closest relative strain	Percentage of identity (%)	Closest homologue accession number
673	M	*Staphylococcus hominis *subsp.* novobiosepticus *	96.4	AB233326.1
		*Staphylococcus haemolyticus strain* 6R-J-5	96.1	EU379304.1
		*Staphylococcus caprae strain* ATCC 35538	97.9	NR024665
		*Bacillus firmus strain* PAN MC15	97.8	HQ285922.1
		*Staphylococcus hominis *subsp.* Hominis* strain FUA 3135	98.3	GQ222404.1
		*Staphylococcus haemolyticus* strain BQN2T-01d	97.0	FJ380980.1
		*Staphylococcus epidermidis* strain BQN1N-02d	97.9	FJ380964.1
		*Kribbia dieselivorans* strain N113	85.9	DQ372707.1
	A	*Staphylococcus saprophyticus* subsp. *saprophyticus *	89.8	AP008934.1
		*Spiroplasma apis* strain ATCC 33834	81.9	GU993267.1

776	M	*Kocuria palustris* isolate PDD-31b-3	91.2	HQ256825.1
		*Bacillus gibsonii* strain FR1_104	97.5	EU373538.1
		*Staphylococcus cohnii* subsp.* urealyticum* strain CK27	95.7	NR037046

843	M	*Staphylococcus hominis* subsp. *novobiosepticus *	97.7	AB233326.1
		*Bacillus gibsonii* strain FR1_104	97.8	EU373538.1
		*Bacillus butanolivorans* strain K9	92.2	EF206294
		*Brevundimonas vesicularis *	98.3	AJ227780
		*Micrococcus luteus* strain Z05	96.2	GU947857.1
		*Staphylococcus haemolyticus* strain BQN2T-01d	97.8	FJ380980
		*Bacillus nealsonii* strain FO-092	95.7	AF234863.1
		*Bacillus firmus* strain PAN MC15	94.1	HQ285922
		*Micrococcus luteus* NCTC 2665	94.0	CP001628
		*Arthrobacter pascens* strain DSM 20545	78.7	NR026191
		*Bacillus muralis* strain REG126	90.6	GQ844961.1
		*Staphylococcus hominis* subsp. *novobiosepticus *	98.3	AB233326.1
	A	*Micrococcus luteus* strain Z05	92.1	GU947857.1
		*Massilia timonae* strain UR/MT95	98.1	NR026014
		*Kocuria rhizophila* strain KL-057	96.8	AY030341.1
		*Staphylococcus epidermidis* strain BQN1N-02d	98.5	FJ380964.1
		*Bacillus galactosidilyticus* strain LMG 17892	94.4	NR025580
		*Bacillus muralis* strain REG126	98.4	GQ844961.1

Facsimile	M	*Mitsuokella multacida *	78.8	X81878.1
		*Staphylococcus hominis *subsp.* novobiosepticus *	97.4	AB233326.1
